# The effect of TCM acupuncture on hot flushes among menopausal women (ACUFLASH) study: A study protocol of an ongoing multi-centre randomised controlled clinical trial

**DOI:** 10.1186/1472-6882-7-6

**Published:** 2007-02-26

**Authors:** Einar K Borud, Terje Alraek, Adrian White, Vinjar Fonnebo, Sameline Grimsgaard

**Affiliations:** 1 National Research Center in Complementary and Alternative Medicine, University of Tromsoe, N-9037 Tromsoe, Norway; 2 Clinical Research Centre, University Hospital of North-Norway, N-9038 Tromsoe, Norway; 3 Department of General Practice and Primary Care, Peninsula Medical School, Plymouth, PL6 8BU, UK

## Abstract

**Background:**

After menopause, 10–20% of all women have nearly intolerable hot flushes. Long term use of hormone replacement therapy involves a health risk, and many women seek alternative strategies to relieve climacteric complaints. Acupuncture is one of the most frequently used complementary therapies in Norway. We designed a study to evaluate whether Traditional Chinese Medicine acupuncture-care together with self-care is more effective than self-care alone to relieve climacteric complaints.

**Methods/Design:**

The study is a multi-centre pragmatic randomised controlled trial with two parallel arms. Participants are postmenopausal women who document ≥7 flushes/24 hours and who are not using hormone replacement therapy or other medication that may influence flushes. According to power calculations 200 women are needed to detect a 50% reduction in flushes, and altogether 286 women will be recruited to allow for a 30% dropout rate.

The treatment group receives 10 sessions of Traditional Chinese Medicine acupuncture-care and self-care; the control group will engage in self-care only. A team of experienced Traditional Chinese Medicine acupuncturists give acupuncture treatments.

**Discussion:**

The study tests acupuncture as a complete treatment package including the therapeutic relationship and expectation. The intervention period lasts for 12 weeks, with follow up at 6 and 12 months. Primary endpoint is change in daily hot flush frequency in the two groups from baseline to 12 weeks; secondary endpoint is health related quality of life, assessed by the Women's Health Questionnaire. We also collect data on Traditional Chinese Medicine diagnoses, and we examine treatment experiences using a qualitative approach. Finally we measure biological variables, to examine potential mechanisms for the effect of acupuncture. The study is funded by The Research Council of Norway.

## Background

After menopause, almost two thirds of women experience hot flushes. One third have symptoms persisting up to five years after natural menopause, and 10–20% find symptoms very distressing [1]. Hormone replacement therapy (HRT) is considered the most effective treatment for hot flushes [2]. However, HRT has other biological effects: it prevents fractures and cancer of the colon [3], but increases the risk of breast cancer [4] and thrombo-embolic disease [5]. Based on these data, the Norwegian Medicines Agency revised the guidelines for prescribing HRT in 2003 [6]. Indications are now only hot flushes and urogenital symptoms. HRT should be used for a short time period, in the lowest possible dose, and other strategies should be considered. The reports of adverse events generated considerable public interest in Norway, and sales figures for systemic HRT have decreased by 45 % since 2001 [7]. Moreover, a Scandinavian study conducted among women treated for breast cancer was terminated when the interim analysis showed that HRT was associated with an increased risk for relapse of the disease [8]. A Norwegian cohort study showed that HRT formulations used in Norway are also associated with an increased risk of breast cancer [9]. The data received considerable public attention and we suppose that they enhanced Norwegian women's interest in low risk strategies to relieve their climacteric complaints.

A large body of data already shows that HRT effectively relieves hot flush activity (frequency and severity) by approximately 80% [2]. Estrogen placebo interventions reduce hot flush activity by 20–30% [10]. Selective serotonin reuptake inhibitors (SSRIs) and selective noradrenaline reuptake inhibitors (SNRIs) reduce the number of hot flushes by 50–60% in the short term in some studies [11]. On this basis it has been advocated that clinically relevant interventions must have the ability to reduce hot flush activity by 50% or more [10].

Women may explore other approaches to manage their menopausal symptoms. These include phyto-estrogens such as soy, herbal remedies such as black cohosh and red clover, or vitamin E. There is a lack of good evidence of their effectiveness, and lack of knowledge of drug interactions and of long-term safety [1]. Commonly recommended lifestyle changes include stress reduction, increased fruit and vegetable intake, reduced caffeine and alcohol intake, smoking cessation, and increased physical exercise. The evidence for these is anecdotal, supported in some cases by epidemiological studies but not by intervention trials [12].

Acupuncture is one of the most frequently used complementary therapies in Norway [13], and is considered safe in the hands of competent practitioners [14]. One small randomised trial (n = 24) conducted in a Swedish university clinic showed that hot flushes decreased by 50% among women who received standardized electroacupuncture [15]. A further study (n = 30) by the same group found a trend in favour of acupuncture compared with sham acupuncture for climacteric symptoms [16]. In a small crossover study (n = 10) in hypertensive postmenopausal women, acupuncture was associated with a significant reduction in 'complaints' though not in blood pressure [17]. An uncontrolled study in 11 participants found a mean reduction in flush score from 4.2 to 1.9 [18]. A small RCT (n = 16) found genuine acupuncture more effective than sham-acupuncture, with a 76% reduction in flushes [19]. An unpublished Norwegian case series (n = 18) suggests that individualized Traditional Chinese Medicine (TCM) acupuncture can reduce hot flushes by 77%. The current data are insufficient to make recommendations regarding acupuncture treatment for hot flushes, but sufficient to justify further research.

Two studies of acupuncture for hot flushes published since the development of this protocol yielded conflicting results: one study of 103 participants found no effect on daily flush frequency [20], whereas a second study of 29 participants found a reduction in severity but not frequency of nocturnal flushes [21].

There is common agreement among acupuncturists that a minimum of six treatment sessions are necessary to evaluate treatment effect, and as many as eight to ten sessions are necessary to establish a maximum effect [22]. Continuation of the effect may require repeated, but less frequent, treatments. If symptoms relapse, as little as two to three treatments can be sufficient to trigger the treatment effect again. An acupuncture session usually costs NOK 300, and accordingly a typical series of treatments costs NOK 3000.

In Norway, most acupuncture practitioners use the TCM approach, and about 500 practitioners belong to the professional organisation, NAFO. A woman who seeks a TCM acupuncture practitioner for hot flushes chooses a treatment strategy rather than a single intervention. The practitioner will most likely focus on climacteric complaints and general well being rather than hot flush activity alone. Treatment involves a comprehensive TCM diagnostic procedure and general life-style advice in addition to individualized acupuncture treatment. Acupuncturists generally consider that these elements interact intentionally with each other during treatment and constitute a treatment package, i.e. "acupuncture-care". If the elements are separated, the treatment will not reflect what TCM acupuncturists consider relevant and good quality acupuncture for a given condition.

## Methods/design

The objective of the study is to address a pragmatic question, to compare the effectiveness of two low risk strategies to relieve climacteric complaints in postmenopausal women who seek alternatives to HRT: is individualized TCM acupuncture-care together with self-care more effective at reducing the frequency and severity of hot flushes compared with self-care alone. The study will test acupuncture as a complete treatment package, i.e. including the therapeutic relationship and expectation, and address the important question of the overall effect of acupuncture-care, which is highly relevant to both postmenopausal women and their health care providers. The limitations of other possible control groups are discussed below. The changes that are likely to be experienced by women undergoing acupuncture in this study will not be fully captured by the quantitative measures used, and therefore the study team will collaborate with other investigators who wish to explore these qualitatively. If the study shows that acupuncture-care has a clinically relevant effect on climacteric complaints, the research group will proceed further and apply for funding to perform a placebo-controlled trial to determine the importance of needle insertion in the overall effect of acupuncture treatment. In addition, a positive result will justify a study of the effect of acupuncture in other groups of patients such as women with hot flushes induced by tamoxifen and men undergoing treatment for prostate cancer.

A sub-study is conducted in 70 women who participate in the Tromsø-arm of the study, to investigate effects of acupuncture-care on biological variables. Evaluations take place at baseline and after 12 weeks. It is hypothesized that acupuncture reduces hot flushes by increasing β-endorphin activity in the hypothalamus [15]. Calcitonin gene-related peptide (CGRP) is a potent vasodilator, and may be a proxy for β-endorphin activity [15]. Plasma concentration of CGRP and neuropeptide Y (NPY) increases during a hot flush [23], and the urine excretion of CGRP is reduced parallel to the reduction of hot flushes after acupuncture treatment [15]. The urine excretion of CGRP and NPY will be measured.

Acupuncture can modulate autonomic nerve system activity [24], and central sympathetic activity is considered to influence hot flush activity [25]. Autonomic nerve system function is assessed by measuring heart rate variability [26]. Bone mass density is measured at baseline and after 12 months, to investigate rate of bone loss among women with climacteric symptoms. Evaluations further include measurements of serum FSH and estradiol at baseline and end of intervention, to confirm that the women are postmenopausal.

Participants are recruited to three centres (Tromsø, Oslo and Bergen), by the use of newspaper advertisements and promotion through media coverage. Informed consent is obtained from all participants before randomisation. See eligibility criteria in table 1.

### Randomization

Participants are stratified by centre and thereafter block randomised (random block size) to additional acupuncture or no additional acupuncture. Staff at study headquarters at University Hospital of North Norway (UNN), which is not connected with the study, prepared the randomisation database and acts as a central randomisation service. Each local coordinator (Tromsø, Oslo and Bergen) obtains distance randomisation of included participants.

### Experimental intervention

Traditional acupuncturists, using diagnostic methods according to the principles of Traditional Chinese Medicine, diagnose TCM syndromes associated with the symptoms of menopausal hot flushes. The acupuncturists meet the criteria for membership in the Norwegian acupuncture society, "Norsk Akupunkturforening" (NAFO), and have at least five years experience of practice. There are four practitioners in Oslo, three in Bergen and three in Tromsø. This will ensure that the study tests the effects of TCM acupuncture-care, not the individual practitioner.

The Delphi technique [27] has been used to establish a consensus between acupuncturists regarding the standardized diagnostic criteria and treatment guidelines, as previously successfully used for acupuncture treatment of lateral epicondylitis [28].

After the initial diagnosis, each participant is treated with points selected according to the syndrome diagnosis. The acupuncturists are free to add individualised points to treat other symptoms related to the menopause (i.e. those included on the WHQ such as depression, anxiety, insomnia), but not unrelated symptoms. In addition, acupuncturists can use moxibustion (warmed needles) if indicated. The acupuncture treatment comprises up to 10 treatment sessions over 12 weeks. This period can be extended for 2 weeks to allow for personal circumstances. The minimum treatment defined 'per protocol' is 6 sessions.

The acupuncture-care group is free to use any over the counter (OTC) drugs and self-provided non-pharmaceutical intervention during the intervention period. In addition, they can use soy, specific dietary supplements and herbal medicines.

### Control intervention

The control group is free to use any OTC drugs and self-provided non-pharmaceutical intervention during the intervention period. In addition, they can use soy, specific dietary supplements and herbal medicines.

Many possible control interventions were considered and rejected for a variety of reasons. Standard HRT has been tested many times and its effect is well known, as is the effect of placebo HRT [2,10]. Acupuncture is expected to be somewhat less effective in relieving hot flushes, and in this study we focus specifically on the increasing number of women who deliberately are seeking an alternative to HRT. At present there is no satisfactory placebo for acupuncture. Needles inserted into the skin avoiding acupuncture points and meridians (sham acupuncture) are likely to have some effect [29], and the blunt needles (Streitberger needles) that have been developed are difficult to use [30]. In addition, acupuncturists are generally uncomfortable using placebo-needles. This attitude is likely to influence the interaction between therapist and study participant and may influence study results. In this study we want to evaluate acupuncture as it is practised in Norway.

### Adjunctive intervention: self care

All participants are given an information leaflet on available self-provided care (e.g. soy, herbs, local oestrogen, physical activity, relaxation techniques to treat menopausal symptoms), and they are free to use any of these.

The information leaflet is specially prepared by the project team and is based on an authoritative book [31] and best current advice [12].

### Effect measures

Primary endpoint is mean number of hot flushes per 24 hours measured over a one-week period. The numbers of hot flushes are recorded in a daily diary, and flushes are scored by severity on a scale from of zero to ten. The diary is administered for two weeks during the qualifying period, and for one week after 4, 8 and 12 weeks of the intervention period.

Secondary endpoint is the Women's Health Questionnaire (WHQ), a validated, self-administered instrument containing 36 questions assessing a wide range of physical and emotional symptoms of women in the postmenopausal period [32]. A Norwegian version is available and has been validated linguistically. A psychometric validation of the WHQ will be performed during the study period. The WHQ is administered at baseline and at 12 weeks.

Baseline assessment includes sociodemographic data collected in a manner parallel to the NOWAC study [33], medical and gynaecological history, previous experience with acupuncture, which other self-provided and therapist-provided interventions they have used earlier to relieve their climacteric complaints, current use of drugs and dietary supplements, level of physical activity, smoking status and level of alcohol consumption.

At the end of interventions, all participants are asked which other self-provided and therapist-provided interventions they have used to relieve their climacteric complaints during the intervention period.

A hot flush diary and the WHQ are administered to participants in the acupuncture group at 6 and 12 months after the baseline evaluation. The follow up-period allows us to investigate the development of symptoms and the use of symptom relieving interventions after the intervention period (natural course of symptoms and choice of treatment).

At each treatment session the acupuncturists ask specifically about, and record, any adverse events that have occurred during or after the last treatment, and adverse event forms are filled in at 4, 8 and 12 weeks. The women are asked specifically about adverse events in the 8- and 12-weekly diaries.

The excretion of CGRP and NPY in early morning and evening urine samples are measured with a radioimmunoassay method [15], to determine any changes during the intervention period. The measurements are related to serum creatinine.

**Figure 1:**
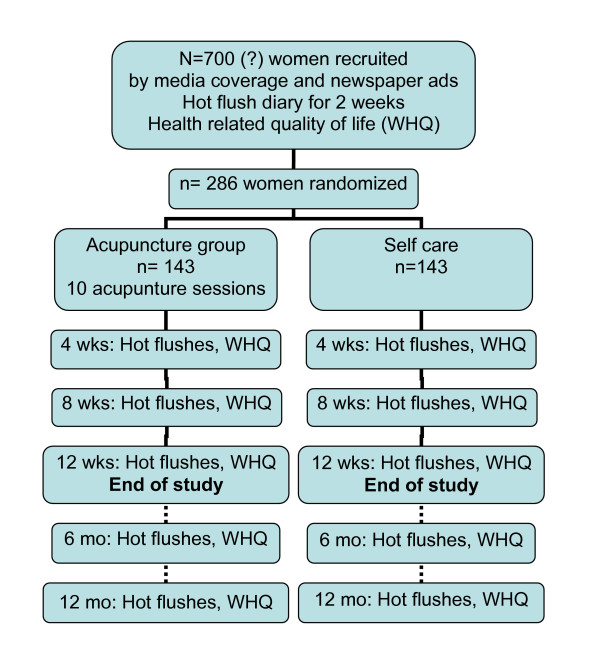
Flow diagram of study design.

Heart rate variability measurements are done at baseline and after 12 weeks. After five minutes rest, a ten minutes registration is done with a Novacor Holter-monitor and Holtersoft Ultima software.  Figure 1 shows a  flow diagram of the study design, and the data collection schedule is  detailed in table 2.

### Statistical power and sample size

Calculations were made in consultation with a professional statistician. The sample size was calculated using NCSS (PASS 2002) programme, using data for hot flush frequency from many previous trials of HRT, herbs and acupuncture. With a baseline daily flush rate of 7.0 (SD 3.0), and assuming that the difference of interest is 20 percentage points (post-treatment flush rates of 3.5 and 4.9 for acupuncture and no acupuncture groups respectively, equivalent to reductions of 50% and 30%, and SD 3.5 for change in flush rate), and employing a two-sided, two-sample t-test for the changes, 100 women in each group were needed to identify a 20% difference with a power of 80% and α-value of 0.05. Assuming a total withdrawal and dropout rate of 30%, we estimated that a total sample size of 286 women is required.

Power calculations for the sub-study of biological variables are based on earlier studies of the CGRP excretion in urine during acupuncture treatment [15]. To identify a supposed 30% difference in CGRP urine excretion with a power of 80% and α-value of 0.05, we need 25–30 participants in each group. To allow for some measurement inaccuracies because CGRP and NYP are to be measured in early morning and evening urine samples, we will use 35 participants in each group.

Data entry is undertaken by the central coordinator, with checking of random 10% samples by the research fellow. Data on primary endpoint are entered by a person not connected to the study team, and who is blinded to treatment allocation.

### Data analysis and handling

All analyses are conducted blinded to group allocation. Missing data are substituted by last value carried forward. The primary analysis is intention-to-treat, comparing mean changes from baseline to end of treatment after 12 weeks in the two groups, using a two-sample t-test. In the presence of meaningful baseline differences, analysis of covariance will be employed. It is anticipated that the data will be somewhat right-skewed, therefore analyses will be checked after appropriate data conversion, and also checked using non-parametric methods. Secondary analyses will compare the changes at different time points. Secondary measures will be analysed using appropriate parametric or non-parametric methods. Subgroup analyses include per-protocol analyses to assess efficacy of acupuncture care, and comparison of changes in hot flush rate among women grouped by TCM syndromes.

The trial headquarters is located at UNN Clinical Research Centre where they have experience in running multicenter studies. All data sheets are rendered anonymous by removing participant names and addresses. The personal information and the index that links trial numbers with individual participants is kept under lock and key in the possession of each local coordinator. Trial number alone identifies all computerised data. To prevent reporting bias, all the participant evaluation forms are administered by the trial headquarters in Tromsø. The participants return the evaluation forms to the trial headquarters in Tromsø.

Participants in either study group who choose to use any therapist-provided care to relieve hot flushes (e.g. massage, homeopathy or any prescribed medication to relieve hot flushes such as systemic estrogen or SSRI's from any source) during the 12 week study period, are followed up with registration of events and included in the intention-to-treat analysis.

### Data and safety monitoring

A Steering Group retains responsibility for quality control and meet on a regular basis throughout the study period. In addition, the Steering Group can be convened as an emergency in the event of a serious adverse event associated with the trial to decide on appropriate action to prevent recurrence. Regular meetings will consider any reported adverse events, protocol violations, the recruitment rate, and any practical issues concerning local coordination, acupuncture practitioners, as well as any issues raised by participants.

The study is performed according to the Helsinki declaration and Good Clinical Practice requirements [34]. The study has been approved by the Norwegian Data Inspectorate and the Norwegian Biobank Registry, and recommended by the Ethics Committee.

## Competing interests

The author(s) declare that they have no competing interests.

## Authors' contributions

EKB is research fellow and main author of this paper. SG conceived of the study, and is the principal investigator. TA is responsible for the design of the acupuncture intervention, and participated in the design of the study. AW is member of the steering group, and gave substantial input to the study design. VF participated in the design of the study, and is member of the steering group. The whole group gave comments on the drafts for this paper.

**Table 1:** Eligibility criteria

**Inclusion criteria**
Postmenopausal (>1 year past last menstruation)
Willing to receive acupuncture
Mean value of ≥7 hot flushes/24 hours during a time period of 7 days

**Exclusion criteria**

Surgical menopause
History of cancer within the past five years
Use of anticoagulant drugs
Heart valve disease
Poorly controlled hypertension
Poorly controlled hypothyroidism
Hyperthyroidism
Poorly controlled diabetes mellitus
Organ transplant
Mental disease
Overt drug or alcohol dependency
Inability to complete study forms
Use of HRT (Wash out period: 8 weeks for systemic and 4 weeks for local use)
Use of SSRI (Wash out period: 8 weeks)

**Table 2:** Data collection schedule

	Qualifying period 2 wks	Baseline Randomisation			End	Follow-up	
Visit number		1			2		3^*1*^
Week number	≤-2	0	4	8	12	26	52

**Participant provided data**							
Hot flush diary	X		X	X	X	X	X
WHQ		X			X	X	X
EQ-5D		X			X	X	X
Sociodemographic variables		X					
Beck depression inventory^*1*^		X			X	X	X
Hopkins symptoms checklist^*2*^		X					
SF36^*2*^		X					
Lifestyle/physical activity		X	X	X	X	X	X
Medical history		X					
Use of health care services ^*3*^		X	X	X	X	X	X
Dietary supplements/herbs		X	X	X	X	X	X
OTC-drugs		X	X	X	X	X	X
Prescribed drugs		X	X	X	X	X	X
Side effects			X	X	X		
**Biological variables **^***1***^							
Sex hormones, creatinine		X			X		
CGRP, NPY		X			X		
Heart rate variability		X			X		
Bone mass density		X					X
**Qualitative data **^***2***^							
Acupuncturist provided data					X		
TCM diagnose		X			X		
Acupoints used		X X	X X X	X X X	X X		
Side effects		X	X X X	X X X	X X		

^*1 *^Subsample of n = 70 women participating in study of biological variables

^*2 *^Subsample of n = 200 women

^*3 *^Including complementary and alternative medicine

## Pre-publication history

The pre-publication history for this paper can be accessed here:


